# Liquefying Laughing Gas

**Published:** 1868-10

**Authors:** 


### LIQUEFYING LAUGHING GAS.

The uniform efficiency and safety of laughing gas as an anæsthetic has prompted *The British Medical Journal* to suggest that a bottle be made strong enough to hold the gas in a liquid form, and of such weight and dimensions that it may be easily carried by the surgeon in his daily rounds. At present it is used by dentists from large gas-bags, into which it is placed as soon as made. Laughing gas is composed, according to the new notation, of two atoms of nitrogen and one of oxygen. These two elements are the principal constituents of common air. Laughing gas or nitrous oxyde can be liquefied under a pressure of 750 pounds per square inch when at the temperature of 45° Fahr. The most convenient and safe receptacle for the liquid would be a brass or copper tube, not more than a foot in length, and of such thickness as to resist a pressure of at least 1500 pounds, or several tubes of the ordinary thickness might be united side by side and made entirely safe.

To get the nitrous oxyde gas into a portable shape, is certainly "a consummation devoutly to be wished."

